# Testosterone and Cholesterol Vasodilation of Rat Aorta Involves L-Type Calcium Channel Inhibition

**DOI:** 10.1155/2010/534184

**Published:** 2010-03-30

**Authors:** E. Álvarez, E. Cairrão, M. Morgado, C. Morais, I. Verde

**Affiliations:** ^1^CICS—Centro de Investigação em Ciências da Saúde, Universidade da Beira Interior, Av. Infante D. Henrique, 6200-506 Covilhã, Portugal; ^2^Centro Hospitalar da Cova da Beira E.P.E., Quinta do Alvito, 6200-251 Covilhã, Portugal

## Abstract

Testosterone has rapid nongenomic vasodilator effects which could be involved in protective cardiovascular actions. Several authors suggested specific mechanisms to explain this effect, but this matter was not clarified yet. We studied the actions of testosterone and cholesterol on endothelium-denuded rat aorta and their effects on the L-type Ca^2+^ current (I_Ca,L_) and potassium current (I_K_). Testosterone (1–100 *μ*M) totally relaxed, in a rapid and concentration-dependent way, the aortic rings contracted by KCl or by (−)-Bay K8644 (BAY). Cholesterol also fully relaxed the contractions induced by KCl. None of the potassium channel antagonists tested (glibenclamide, tetraethylammonium and 4-aminopyridine) modified significantly the relaxant effect of testosterone. The antagonist of classic testosterone receptors, flutamide, did not modify the vasorelaxant effect of testosterone. Furthermore, testosterone and cholesterol inhibited either basal and BAY-stimulated I_Ca,L_ in A7r5 cells and they have no effects on I_K_. In summary, our results demonstrate that cholesterol and testosterone relax rat aorta by inhibiting LTCC. This effect of testosterone is not mediated by the classic hormone receptor or by potassium channel activation. These results suggest that the vasodilator mechanism of cholesterol and testosterone is the same.

## 1. Introduction

Gender differences in the incidence of cardiovascular health problems were attributed to different sex hormonal patterns found in women and men. Some studies and clinical trials suggested a direct modulation of vascular function by both female and male sex hormones [1]. Testosterone was associated with negative effects on the cardiovascular system, such as increased cardiovascular disease risk, thrombosis, cardiac hypertrophy, and suspected proatherogenic effects [2, 3]. However, more recent studies illustrated that testosterone has some beneficial cardiovascular effects and several epidemiological studies also indicated that patients with cardiovascular diseases have low levels of testosterone [4–7]. On the other hand, high cholesterolemia was related with the increase in cardiovascular diseases. Some studies attempting to link hypercholesterolemia with abnormal vascular smooth muscle (SMC) contractions have focused on the endothelium. 

 In the last years, vasodilatation induced by testosterone has been shown in different vessels from different species [8, 9]. This vasodilatation is not attenuated by pretreatment with the classic androgen receptor blocker flutamide [10–12] and nongenomic testosterone analogues have also been shown to elicit greater vasodilatation than genomic-acting analogues [12, 13]. On the other hand, studies performed with different rat vessels showed that removal of the endothelium slight reduces the testosterone relaxant effect in aorta [13] and mesenteric artery [11, 14]. However, Yue et al. (1995) indicated that testosterone induces endothelium-independent relaxation in isolated coronary artery and aorta from rabbit [12]. Furthermore, testosterone-induced relaxation of porcine coronary arteries was associated with accumulation of cGMP by an endothelium-independent mechanism [15]. 

 Concerning the testosterone modulation of membrane ionic fluxes in vascular smooth muscle cells, it was suggested that in rat aorta LTCC are inhibited by physiological concentrations of this hormone [16, 17], while T-type currents are only blocked at higher concentrations [17]. On the other hand, the functional implication of potassium channels opening in the testosterone-induced vasodilatation of different arteries from distinct species has been proposed [10, 11, 18–20]. Activation of potassium channels in vascular smooth muscle may induce hyperpolarization of plasma membrane, which leads to close LTCC and vascular relaxation. Honda et al. suggested that this mechanism could be more relevant in situations of systemic hypertension [18]. 

 The effects of cholesterol initially were thought to be mediated by the endothelium, and some authors suggested that cholesterol decreases the production and/or the availability of endothelium-derived factors, mainly NO, leading to endothelial dysfunction and abnormal vascular reactivity [21]. Recently, it was suggested that cholesterol: increases vascular sensitivity by increasing Ca^2+^ permeability [22], increases calcium sensitization through the Rho-kinase (ROCK)-mediated pathway [21], influences vascular reactivity to endothelin-1 and 5-HT [22, 23], decreases the expression of LTCC [24], and regulates the expression levels of specific inward rectifier and ATP-sensitive potassium channel subtypes [25]. On the other hand, farnesol, a nonsterol mevalonate derivative from the cholesterol synthesis pathway, was reported to inhibit L-type calcium channels (LTCC) in vascular smooth muscle cells [26, 27] and it also induces relaxation of contracted rat aortic and human mesenteric arteries [28]. 

In summary, testosterone has vascular nongenomic actions which include vasodilatation, and the effect of cholesterol at this level is uncertain. The vasodilatation induced by testosterone could involve the modulation of several ionic channels. The purpose of this study was to analyse the effects of testosterone and cholesterol in rat aortic smooth muscle comparing the mechanisms implicated in each case. The effect of testosterone and cholesterol on contracted endothelium-denuded rat aorta was analysed. The whole cell configuration of the patch-clamp technique was used to analyse the effects of testosterone and cholesterol on the calcium and potassium currents in A7r5 cells.

## 2. Methods

### 2.1. Rat Aorta Contractility Experiments

Male adult Wistar rats (Charles-River, Barcelona, Spain) weighing 400–500 g were housed and acclimatized for at least one week before performing the experiments in appropriate laboratory installations with light cycles of 12 hours light: 12 hours dark and food and water *ad libitum*. The rats were used in accordance with the European regulations about protection of animals (Directive 86/609) and the Guide for the Care and Use of Laboratory Animals promulgated by the US National Institutes of Health (NIH Publication No. 85–23, revised 1996).

 The rats were sacrificed by decapitation. After thoracotomy, the aortas were obtained, placed in a thermostatized (37°C) Krebs modified solution and the fat and connective tissue were cleaned. Vascular endothelium was mechanically removed by gentle rubbing with a cotton bud introduced through the arterial lumen. The artery rings were placed in an organ bath (LE01.004, Letica) containing Krebs-bicarbonate solution at 37°C continuously gassed with carbogen. The composition of the Krebs' modified solution was (mM): NaCl 119, KCl 5, CaCl_2_·2H_2_O 0.5, MgSO_4_·7H_2_O 1.2, KH_2_PO_4_ 1.2, NaHCO_3_ 25, EDTA-Na_2_ 0.03, L-(+)-ascorbic acid 0.6 and glucose 11 (pH 7.4). The rings were suspended by two parallel stainless steel wires and tension measurement was performed using isometric transducers (TRI201, Panlab SA, Spain), amplifier (ML118/D Quad Bridge, ADInstruments), interface PowerLab/4SP (ML750, ADInstruments), and computerised system with Chart5 PowerLab software (ADInstruments). During the resting periods, the organ bath solution was changed every 15 minutes.

 Initially, the rings were equilibrated for 60 minutes until a resting tension of 1.0 g. After the equilibration period, aortic rings were firstly contracted with high isosmotic KCl concentrations (60 mM) and the absence of endothelium functionality was confirmed by the lack of relaxant response to acetylcholine (1 *μ*M). After that, the arteries were washed and allowed to recuperate for at least 45 minutes before the next induced contraction. The rings were contracted using KCl (60 mM) or (−)-Bay K 8644 (BAY; 0.1 *μ*M) and vasorelaxation induced by testosterone (1–100 *μ*M) on these contractions was analysed. 

 Aortic rings were contracted with BAY (0.1 *μ*M) after increasing to 10 mM the KCl concentration in the Krebs solution. The Krebs KCl concentration was increased to 10 mM to facilitate the opening of Ca^2+^ channels by Bay K8644. The effect of cholesterol (1–100 *μ*M) in the artery rings contracted with KCl (60 mM) was also analysed. In some experiments, the involvement of the classical hormonal receptors in the vasorelaxant effects of testosterone was studied using flutamide, a specific antagonist for the classical hormonal receptor. In these cases, after contraction, the arteries were incubated 15 minutes with flutamide (10 *μ*M) and the effect of testosterone in the presence of this antagonist was analysed. To determine the role of potassium channels activation in testosterone effects, several potassium channel inhibitors were used in some experiments: tetraethylammonium (TEA; 1 mM), an inhibitor of BK_Ca_; glibenclamide (10 *μ*M), an inhibitor of K_ATP_; and 4-aminopyridine (4-AP; 1 mM), an inhibitor of K_V_. In these cases, after contraction, the arteries were incubated 15 minutes with the potassium channel inhibitors and the effect of testosterone in presence of these drugs was analysed. Control experiments with ethanol, the vehicle used to dissolve the drugs, were always performed.

### 2.2. Cell Culture of Vascular Smooth Muscle Cells

The A7r5 cell line, used in this study, is a commercial vascular smooth muscle cell line obtained from embryonic rata aorta (Promochem, Spain). The cells were grown in culture medium Dulbecco's Modified Eagle's Medium/Nutrient Mixture F-12 Hams (DMEF-F12; Sigma-Aldrich, Portugal) supplemented with NaHCO3 (1.2 g/L), L-ascorbic acid (20 mg/L; Sigma-Aldrich), bovine serum albumin (0.5%; Sigma-Aldrich), heat-inactivated foetal bovine serum (FBS; 10%; Biochrom), and a mixture of penicillin (100 u/mL), streptomycin (100 *μ*g/mL), and amphotericin B (250 ng/mL) (Sigma-Aldrich). The cells were kept in culture at 37°C in a humidified atmosphere with 5% CO_2_ in air. After confluence, the cells were placed in culture medium without FBS (FBS-free culture medium) for 24–48 hours. Trypsinization was made using a solution of trypsin (0.3%) in Ca^2+^-Mg^2+^-free phosphate buffered solution with EDTA (0.025%). Subsequently, the cells were kept at 4°C in FBS-free medium until the realisation of the electrophysiological experiments.

### 2.3. Electrophysiological Experiments

The whole cell configuration of patch clamp technique was used to analyse the L-type calcium current (I_Ca,L_) and the potassium current (I_K_). 

 To analyse the I_Ca,L_, the control external solution contained (mM): NaCl 124.0, CaCl_2_ 5.0, HEPES 5.0, tetraethylammonium sodium salt (TEA) 10.0, KCl 4.7 and glucose 6.0, pH 7.4 adjusted with NaOH. Patch electrodes (2–4 M*Ω*) were filled with internal solution (mM): CsCl 119.8, CaCl_2_ 0.06, MgCl_2_ 4.0, Na-ATP 3.1, Na-GTP 0.4, EGTA 5.0, HEPES 10.0 and TEA 10.0, pH 7.3 adjusted with CsOH. The presence of Cs^+^ instead of K^+^ in the solutions blocked the potassium currents. The cells were maintained at a holding potential of –80 mV and routinely depolarised every 8 s to 0 mV test potential during 500 ms to measure I_Ca,L_. 

 To analyse the I_K_, the control external solution contained (mM): NaCl 134.3, CaCl_2_ 1.0, HEPES 5.0, KCl 5.4, and glucose 6.0, pH 7.4 adjusted with NaOH. Patch electrodes (2–4 M*Ω*) were filled with internal solution (mM): KCl 125.0, MgCl_2_ 1.0, Na-ATP 5.0, Na-GTP 0.5, EGTA 0.1, HEPES 20.0, and glucose 10.0, pH 7.3 adjusted with KOH. For I_K_ analysis, we used the same holding potential and depolarizations to 60 mV for 300 ms were performed every 8 s. 

 Basal I_Ca,L_ and I_K_ were measured 3–5 minutes after patch break to allow the equilibration between pipette and intracellular solutions. Currents were not compensated for capacitance and leak currents. All experiments were done at room temperature (21–25°C) and the temperature did not vary by more than 1°C in a given experiment. The cells were voltage clamped using the patch-clamp amplifier Axopatch 200B (Axon instruments, USA). Currents were sampled at a frequency of 10 kHz and filtered at 0.1 kHz using the analog-digital interface Digidata 1322A (Axon Instruments, USA) connected to a compatible computer with the Pclamp8 software (Axon Instruments, USA). The external solution was applied to the cell proximity by placing the cell at the opening of a 250 *μ*m inner diameter capillary tube flowing at a rate of 20 *μ*L/minutes. The basal and BAY-stimulated (10 nM) I_Ca,L_ were studied in the presence of different concentrations of testosterone (1–100 *μ*M) and of cholesterol (1–100 *μ*M) dissolved in the external solution.

### 2.4. Drugs

All drugs and chemicals were purchased from Sigma-Aldrich Química (Sintra, Portugal), except 4-aminopyridine that was purchased from Biogen Cientifica (Madrid, Spain). 

 Flutamide, (−)-Bay K 8644 (BAY), nifedipine (NIF), cholesterol, and testosterone were initially dissolved in ethanol. 4-Aminopyridine (4-AP), glibenclamide, apamin and tetraethylammonium sodium salt (TEA) were initially dissolved in deionised water. Appropriate dilutions in Krebs modified solution or in the corresponding electrophysiology external solution were prepared every day before the experiment. Final concentration of ethanol never exceeded 0.1% in the experiments.

### 2.5. Statistical Analysis

Statistical treatment of data was performed using the SigmaStat Statistical Analysis System, version 1.00 (1992). Results are expressed as mean ± SEM of *n* experiments. In the contractility experiments *n *indicates the number of rings used, that were obtained from at least 3 animals. In the electrophysiological experiments *n* is the number of cells analysed. Comparison among multiple groups was analysed by using a one-way ANOVA followed by Dunnet's post hoc test to determine significant differences among the means. Comparison between two groups was analysed by using Students *t*-test. Probability levels lower than 5% were considered significant (*P* < .05). 

 In the contractility experiments, the relaxant responses induced by testosterone and cholesterol are expressed as a percent of the maximal contraction (*E*_max _=100%) produced by the corresponding vasoconstrictor agent. In these experiments, sigmoidal concentration-response curves for the vasorelaxant effects were fitted and IC_50_ values (i.e., concentrations inducing 50% of relaxation) were estimated for KCl- or BAY-induced contractions. The antagonist of classical androgen receptors, flutamide, relaxed by itself the arteries contracted by KCl, and in this case the maximal effect used to perform the concentration-response curves was the tension obtained in presence of flutamide. 

 The I_Ca,L_ amplitudes were automatically calculated between the maximum current peak and the stable current plateau near the final of the every 8 s pulse. The I_Ca,L_ variations induced by the different drugs used are expressed as a percent of the basal or BAY-stimulated I_Ca,L_. The I_K_ variations are expressed as a percent of the basal I_K_ obtained by depolarization in the absence of any drug.

## 3. Results

### 3.1. Vasorelaxant Effects of Testosterone and Cholesterol in Rat Aorta

The rat aortic rings without endothelium were contracted by depolarisation with isosmotic KCl (60 mM) solution and by the calcium channel opener BAY (0.1 *μ*M). Maximal contractions elicited by KCl and BAY, 1174.4 ± 27.5 mg (*n* = 54) and 1293.6 ± 77.1 mg (*n* = 18), respectively, were not significantly different (*P* > .05, Student's *t*-test). These contractile effects were reversible after washing out with Krebs solution.

**Figure 1:**
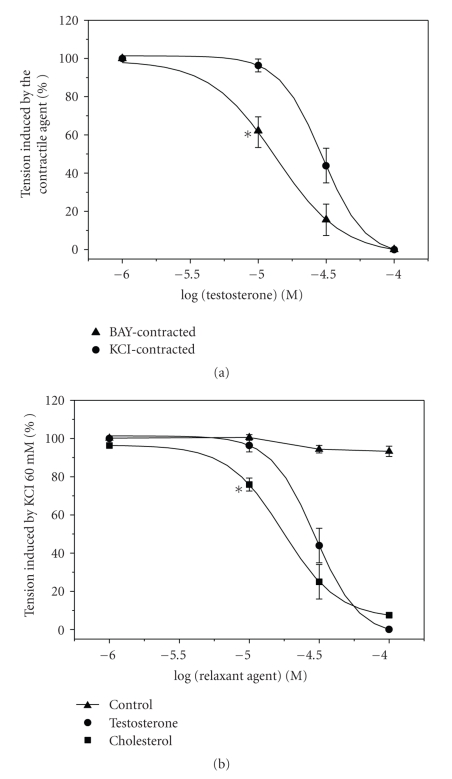
Vasorelaxant effect of testosterone and cholesterol on rat aorta. (a): Effect of cumulative concentrations of testosterone (1–100 *μ*M) on contractions elicited by KCl (60 Mm) and BAY (0.1 *μ*M). (b): Effect of cumulative concentrations of testosterone and cholesterol (1–100 *μ*M) on contractions elicited by KCl (60 mM). Control curve with the effect of the steroids solvent used (ethanol) is also shown. Each point represents the mean value and the vertical lines indicate SEM of at least 5 experiments. *P < .05 versus testosterone effect on KCl contractions, Student's *t*-test.

 After obtaining stable contraction with KCl and BAY, the cumulative addition of testosterone (1–100 *μ*M) fully relaxed (100%) in both cases these contractions in a concentration dependent manner (Figure 1(a)). The vasorelaxation induced by each concentration of testosterone was observed after 10–15 minutes. The use of testosterone did not injure the contractility properties of the artery because, after washing out, a second administration of the contractile agents elicited a similar contraction than the previous one (*P* > .05, data not shown). The maximal relaxation induced by testosterone was similar in arteries contracted by KCl or BAY (Figure 1(a)). However, the IC_50_ obtained from KCl-contracted arteries is significantly bigger than the obtained from BAY-contracted arteries (Table 1).

 Cholesterol (1–100 *μ*M) also induced concentration-dependent vasorelaxations of the rat aortic rings contracted by KCl. The maximal relaxing effect of cholesterol was similar than the elicited by testosterone (*P* > .05) (Figure 1(b)). However, the IC_50_ for testosterone (29.88 ± 1.12; *n* = 5) was significantly bigger than the obtained for cholesterol (19.10 ± 2.58; *n* = 6) (*P* < .05, Student's *t*-test).

 Ethanol, the vehicle used to dissolve testosterone and cholesterol, did not have significant relaxant effect at the concentrations used (Figure 1).

 The vasorelaxant effects of testosterone in rat aorta could be mediated by the activation of the classical intracellular receptors. To test this possibility, the effect of flutamide (10 *μ*M) on the testosterone vasorelaxations was analysed. Initially, after contraction by KCl, the artery rings were exposed for 15 minutes to flutamide, which caused a significant relaxation on rat aortic rings (54.8 ± 2.7%). However, flutamide did not affect the vasorelaxant effects of testosterone, because the IC_50_ values obtained in the absence and in the presence of this antagonist were similar (*P* > .05, Student's *t*-test; Table 1). 

**Figure 2:**
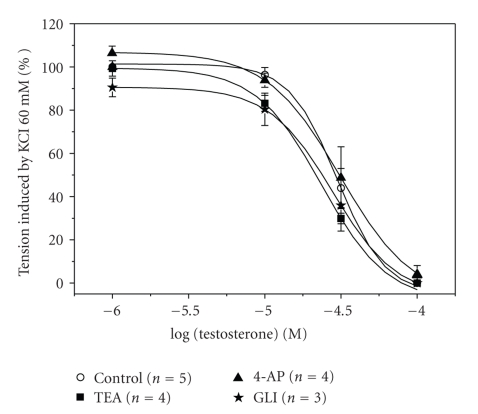
Effect of potassium channel inhibitors on rat aorta relaxation induced by testosterone. Effect of increasing concentrations of testosterone (1–100 *μ*M) in endothelium-denuded rat aortic rings contracted with KCl (60 mM) in presence or absence of the potassium channel inhibitors glibenclamide (GLI; 10 *μ*M), 4-aminopyridine (4-AP; 1 mM), or tetraethylammonium (TEA; 1 mM). Each point represents the mean value ± SEM (indicated in vertical bars) from the number of experiments shown in brackets.

 The effects of inhibitors of three different potassium channels (glibenclamide, 4-AP, and TEA) were also investigated in order to analyse the involvement of these channels in the relaxant mechanism of testosterone. The presence of glibenclamide, 4-AP or TEA, did not have a significant effect on the contraction induced by KCl (data not show) and did not modify significantly the relaxant effect of testosterone (Figure 2) (*P* > .05, one-way ANOVA with Dunnet's post hoc test). The IC_50_ values calculated for testosterone in the presence of anyone of the K^+^ channel inhibitors did not differ significantly from the IC_50_ values calculated in the absence of the blockers (*P* > .05; Table 1).

### 3.2. Effects of Testosterone and Cholesterol on I_Ca,L_ in A7r5 Cells

**Figure 3:**
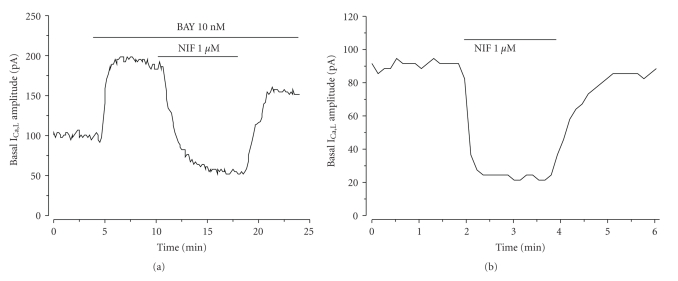
Effect of nifedipine and BAY on I_Ca,L_ amplitude in A7r5 cells. Original records of I_Ca,L_ measured in Patch-clamp experiments showing that: BAY (10 nM) stimulates I_Ca,L_ and nifedipine (NIF; 1 *μ*M) inhibits the BAY stimulation (a); Nifedipine(1 *μ*M) directly inhibits the basal I_Ca,L_ (b).

The whole-cell patch clamp technique was used to analyse calcium current through the LTCC (I_Ca,L_) in A7r5 cells. The mean value of basal I_Ca,L_ density was of 0.93 ± 0.05 pA/pF (*n* = 91). The application of BAY (10 nM; specific stimulator of LTCC) significantly stimulated the calcium current on 74.8 ± 5.7% (*n *= 36) above the basal level. On the contrary, nifedipine (1 *μ*M; LTCC inhibitor) significantly reduced the current until a level of 20.6 ± 2.2% (*n *= 8) of the basal current (*P* < .05). Even so, the effects of BAY and/or nifedipine were completely reversible upon washout of the drug (Figure 3). These results indicate that the current analysed is a LTCC current (I_Ca,L_). Figure 3 shows the time course of two experiments in which BAY (10 nM) stimulates basal I_Ca,L_ (Figure 3(a)) and nifedipine (1 *μ*M) inhibited both BAY-stimulated and basal (Figures 3(a) and 3(b), resp.).

**Figure 4:**
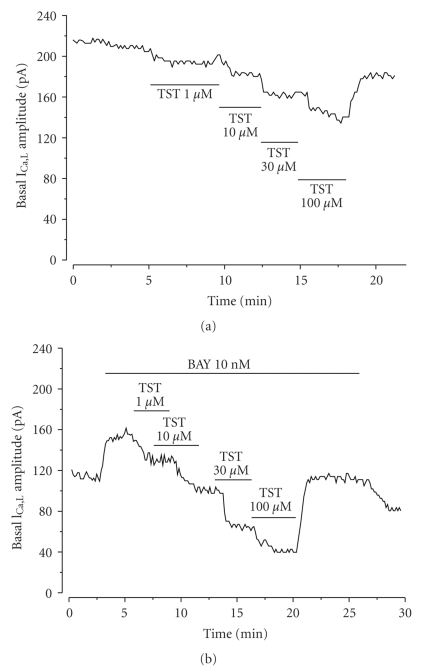
Effect of testosterone on I_Ca,L_ amplitude in A7r5 cells. Original records of I_Ca,L_ measured in Patch-clamp experiments showing that increasing concentrations of testosterone (TST; 10-100 *μ*M) inhibit basal I_Ca,L_ (a) and BAY-stimulated I_Ca,L_ (b).

**Figure 5:**
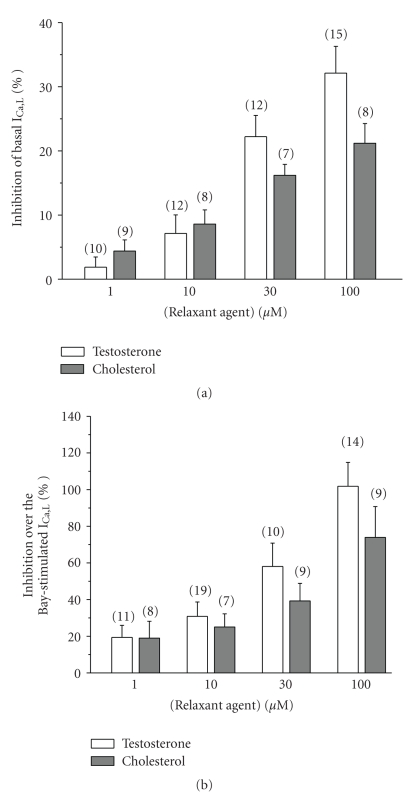
Effects of testosterone and cholesterol on I_Ca,L_ in A7r5 cells. Different concentrations (1–100 *μ*M) of testosterone and cholesterol inhibit basal I_Ca,L_ (a) and BAY-stimulated (10 nM) I_Ca,L_ (b). Each column represents the mean value ± SEM (indicated in vertical bars), in percent of the basal (a) or BAY-stimulated (b) I_Ca,L_ from the number of experiments shown in brackets.

 Like a proposed vasodilatatory mechanism of testosterone is the inhibition of LTCC, we tested the effect of this steroid on I_Ca,L_.  Figure 4(a) shows a typical experiment in which different concentrations (1–100 *μ*M) of testosterone inhibited the basal I_Ca,L_ in a reversible way.  Figure 5(a) summarises the results of this type of experiments in which testosterone at different concentrations (1, 10, 30 and 100 *μ*M) inhibited basal I_Ca,L_ in a concentration-dependent manner. The effect of cholesterol on the basal I_Ca,L_ was also analysed (Figure 5(a)), and like testosterone, cholesterol (1–100 *μ*M) inhibited the basal I_Ca,L_. Furthermore, cholesterol seems to have similareffects that of testosteroneon basal I_Ca,L_ (*P* > .05, Student's *t*-test), even if the testosterone effects are bigger.

 To further characterize the inhibitory effects of testosterone on vascular LTCC, we analyse their effect on the I_Ca,L_ stimulated by the LTCC agonist BAY.  Figure 4(b) shows a typical experiment in which different concentrations (1–100 *μ*M) of testosterone reversibly inhibited the I_Ca,L_ stimulated by BAY (10 nM). The inhibitory effect of testosterone on BAY-stimulated I_Ca,L_ was dependent on the concentration. The 100 *μ*M concentration testosterone completely inhibited the stimulation of BAY reducing the I_Ca,L_ below the basal I_Ca,L_ levels (Figure 5(b)). To further characterize the inhibitory effects of cholesterol on vascular LTCC, we also analyse their effect on the I_Ca,L_ stimulated by BAY. Cholesterol inhibited BAY-stimulated I_Ca,L_. The maximal concentration of cholesterol used inhibited on 73.9 ± 16.8% the BAY-stimulated I_Ca,L_ (Figure 5(b)). Ethanol (0.001–0.1%), the vehicle used to dissolve testosterone and cholesterol, did not affect basal or stimulated I_Ca,L_ (data not shown). The effect of testosterone and cholesterol on BAY-stimulated I_Ca,L_ is not significantly different (*P* > .05, Student's *t*-test), even if the cholesterol effects are lower.

### 3.3. Effects of Testosterone and Cholesterol on I_K_ in A7r5 Cells

**Figure 6:**
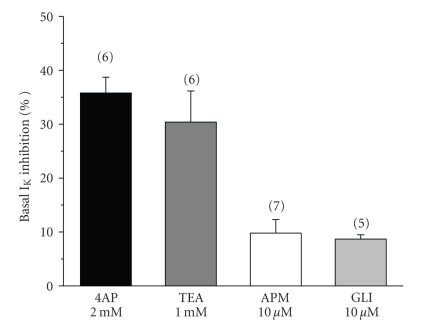
Effect of different potassium channel blockers on I_K_ in A7r5 cells. The bars represent the effect on I_K_ of the following potassium channel blockers: the K_V_ channel blocker 4-aminopyridine (4-AP; 2 mM); the BK_Ca_ channel blocker tetraethylammonium (TEA; 1 mM); the low-conductance K_Ca_ channels blocker apamin (APM, 10 *μ*M); and the K_ATP_ channel blocker glibenclamide (GLI; 10 *μ*M). Each column represents the mean value ± SEM (indicated in vertical bars), in percent of the inhibition of I_K_ from the number of experiments shown in brackets.

The whole-cell patch clamp technique was used to analyse potassium current (I_K_) in A7r5 cells. The mean value of basal I_K_ density was of 9.1 ± 1.4 pA/pF (*n* = 34). In order to determine the types of potassium channels that were responsible for the total current measured, we used selective blockers of different channels. The K_V_ channel blocker 4-AP reduced basal I_K_ on 35.8 ± 2.9% at +60 mV. TEA (1 mM), which is used as a BK_Ca_ channel blocker, reduced net current by 30.4 ± 5.7% at +60 mV (Figure 6). We also tested the presence of the low-conductance K_Ca_ channels using the selective blocker apamin (10 *μ*M), which induced a small reduction on the basal I_K_ (9.8 ± 2.5%). Glibenclamide, usually used as a K_ATP_ channel blocker, also induced a small reduction on the I_K_ (8.7 ± 0.8%, *n* = 5) (Figure 6). The effects of the potassium channels blockers used were completely reversible upon washout of the drug. Thus, our data suggest that the potassium current measured is mainly constituted by potassium exit through K_V_ and BK_Ca_ channels. 

 In order to further characterize the vascular relaxant mechanism of testosterone, the effects of this steroid on A7r5 I_K_ were analyzed. The results show that different concentrations (1–100 *μ*M) of testosterone did not inhibit the I_K_ current (Table 2). Similar results were observed with different concentrations of cholesterol (Table 2).

## 4. Discussion

In the present study, we analyzed the effect of testosterone and cholesterol on endothelium-denuded rat aorta contracted arteries and on the I_Ca,L_ and I_K_ measured by whole cell voltage-clamp in A7r5 cells. 

 The testosterone relaxant effect was previously observed by other authors working with rat aorta [14, 18, 29, 30] and other arteries such as coronary artery from dogs [31] and from humans [32], or human umbilical artery [10]. The vasorelaxant effect of testosterone in rat denuded aortic rings contracted with KCl was concentration-dependent and the maximal relaxation effect obtained was 100%, data that are in agreement with the obtained by Tep-areenan et al. [14]. Several authors suggested that vasorelaxant effect is partially dependent of the endothelium [11, 13, 14, 18]. Our data show that, regardless of the endothelium role, the effect of testosterone is induced in absence of the endothelium, in agreement with other authors [12, 15, 30, 33]. We show that testosterone fully relaxed the arteries contracted either by KCl or BAY, although the IC_50_ was bigger for KCl contracted arteries. High extracellular KCl concentrations induce plasma membrane depolarization. This depolarization can activate voltage-dependent channels, among them LTCCwhoseopening increases intracellular calcium levels and muscle contraction. BAY directly and specifically opens LTCC, equally inducing vascular smooth muscle contraction by intracellular calcium increase. Thus, these results show that testosterone inhibits KCl- and BAY-induced contractions and point to LTCC inhibition as a cause of this effect, as previously suggested by other authors [34]. To confirm this, we performed patch clamp studies in A7r5 to analyse the testosterone effect on the activity of LTCC. BAY, a known agonist of this type of channels, clearly stimulates the basal I_Ca,L_, and nifedipine, a selective antagonist of LTCC, significantly blocked either basal or BAY-stimulated calcium current. These data confirm that calcium current measured is due to calcium entry through LTCC. Our results also show a rapid concentration-dependent inhibitory effect of testosterone on basal I_Ca,L_. Other authors suggested previously that, in A7r5 cells, testosterone inhibits LTCC [16, 17] and T-type calcium channels [17]. Several authors have suggested that the vasorelaxation induced by androgens in rat aorta may be induced by non LTCC because the relaxation induced by these steroids is minor when contraction is induced by BAY, than in KCl or noradrenaline induced contractions [35]. However, our results show that the contraction induced by BAY was abolished by testosterone and, for the first time, we showed that testosterone inhibits BAY-stimulated I_Ca,L_, confirming the inhibitory properties of testosterone on rat aorta LTCC.

 The concentrations of testosterone required to induce in vitro vasodilatation were supraphysiological. In general, wide-ranging discrepancies exist between the concentrations of testosterone required to produce effects in vivo and in vitro. In general the androgen concentrations needed to induce a relaxing effect are supraphysiological [8, 12, 35, 36]. Concerning the effects on ionic channels, previous studies with vascular smooth muscle cells showed discrepancies in the testosterone concentration needed to observe these effects. In A7r5 cells, Scragg et al. observed that testosterone inhibits LTCC and T-type calcium channels at supraphysiological concentrations [17]. In the same cells, Hall et al. observed that nanomolar concentrations of testosterone inhibit LTCC [16]. In HEK293 cells transfected with the alpha1C subunit of the human cardiovascular LTCC, it was shown that concentrations of testosterone from 0.1 to 100 *μ*M inhibit these channels [37]. In fresh rat aortic myocytes, Montano et al. also observed that physiological concentrations of testosterone inhibit LTCC [36]. As other lipophilic substances, testosterone is transported by plasma proteins, like the sex hormone binding globulin (SHBG). The recent identification of membrane receptors for SHBG in some cell types has prompted the suggestion that the hormone-globulin complex may be able to elicit cellular responses or that SHBG might be responsible for the correct orientation of testosterone within the target in cell membrane [38]. If the SHBG is involved in testosterone presentation to cell membrane proteins, the absence of this signalling mechanism in our experiments may contribute to the marked loss of potency in these studies. Nevertheless, the elevated concentrations needed seem to be related with the solubility of the steroids because in our experimental conditions there are not steroid binding proteins.

 The inhibitory effect of testosterone was rapid and reversible, and this effect disappeared after drug washing. These data suggest that testosterone effect is mediated by a nongenomic pathway. Previously, some authors described the existence of a nongenomic mechanism induced by sex steroids which regulates the vascular tone [39]. Besides, the existence of an unknown receptor, not yet identified and placed in the cell surface or in the intracellular space, was suggested [38, 40]. Also, testosterone could block LTCC by direct binding to the channels [41]. Moreover, recently Scragg et al. observed that the LTCC mutation at the nifedipine binding site results in the loss of the testosterone vasorelaxation effect [37]. On the other hand, other authors showed that increase of cyclic nucleotide levels was associated with the vasodilator effects of testosterone in porcine coronary myocytes and in rat aortic myocytes [15, 36], suggesting an interaction of testosterone with the cyclic nucleotide pathway. 

 In contrast with the hypothesis about calcium channel inhibition, other investigators reported that the vasodilator effect of testosterone is due to the stimulation of potassium channels [13, 18]. The activation of potassium channels in vascular smooth muscle may induce repolarization and closing of LTCC, contributing to vascular relaxation. The bibliographic data about the role of different potassium channels in the vasorelaxant effect of testosterone are controversial. Activation of BK_Ca_ channels has been implicated in the action of testosterone in rat mesenteric arterial bed [11, 19], in porcine coronary myocytes [15], in human internal mammary artery [20], and in human umbilical artery [10]. The participation of K_V_ channels in the testosterone relaxing effect was reported in rat aorta [13], rabbit coronary arteries [19], and human umbilical artery [10]. Finally, K_ATP_ channel mediates the testosterone vasodilation of rat aorta [18], human radial artery [42], and human corpus cavernosum [43]. We used different types of potassium channels inhibitors, to test the involvement of these channels in the testosterone effect. TEA, glibenclamide, and 4-AP did not significantly modify the vasorelaxant effects of testosterone suggesting that potassium channel opening is not involved in the rat aorta vasodilatation induced by testosterone. To further investigate the effect of testosterone on potassium channels, we performed patch clamp studies in A7r5. The current measured was significantly inhibited by the K_V_ channel blocker (4-AP) and by the BK_ca_ channel blocker (TEA), but the low-conductance K_Ca_ channel blocker (apamin) and the K_ATP_ channel blocker (glibenclamide) only have a small effect in potassium currents. Thus, our results showed that the potassium current in A7r5 cells is mainly due to K_V_ and BK_ca_. On the other hand, our data also show that testosterone failed to stimulate I_K_ in A7r5 cells, confirming the contractility data, and demonstrating that potassium channels are not implicated in the testosterone vasorelaxant effect in rat aorta. These data agree with those obtained in pig prostatic small arteries [33] and in rat thoracic aorta [36]. In fact, the effect of testosterone on potassium channels was never demonstrated in A7r5 cells. 

 Several authors have suggested that testosterone-induced vasodilatation is not attenuated either by pretreatment with the classic androgen receptor blocker flutamide [10–12]. Also, polar, nonpermeable testosterone analogues have been shown to elicit greater vasodilatation than nonpolar, permeable analogues [13]. Besides, testosterone-mediated vasodilatation is maintained in vessels with androgen receptor deficiency [41]. Thus, the effect seems to be independent of the classical genomic signalling pathway and this effect is mediated by a different signalling pathway or an unknown receptor. Concerning the rat aorta, some authors observed that flutamide did not inhibit testosterone-induced vasorelaxation [12, 14]. Tep-areenan et al. also demonstrate that mifepristone (an unspecific steroid receptor antagonist) did not inhibit the testosterone effect [14]. Our data also show that the blockage of the intracellular testosterone receptor did not modify the vasorelaxant action of testosterone, confirming that the testosterone effect is independent of the androgen receptor. In contrast, Murphy and Khalil showed that the testosterone vasorelaxant effect in pig coronary artery was inhibited by flutamide [44]. On the other hand, our data show that flutamide elicited an unexpected and direct relaxation of KCl-contracted arteries. In this sense, Iliescu et al. have previously shown a vasorelaxation effect induced by flutamide in rat aorta, and suggested that this effect is independent of the nuclear receptor activation and involves activation of the NO-cGMP pathway [45]. Ba et al. also observed this effect in rat arteries and a bigger flutamide relaxation in arteries from males than from females, suggesting a sex-dependent mechanism [46].

 Several investigators observed that other sex steroids have the same vasodilator effect than testosterone [47, 48]. Also, some of the mechanisms proposed to explain the vasodilator effects of these steroids aresimilar to thatproposed for testosterone, such as inhibition of calcium entry by 17beta-estradiol and progesterone in pig coronary arteries [48], or inhibition of LTCC by estrogens in rat aorta [49]. These data could suggest an unspecific effect of this group of substances. In fact, farnesol, a nonsterol mevalonate derivative and intermediate of the cholesterol synthesis pathway, was also reported to inhibit LTCC of vascular smooth muscle cells [26, 27] and also induces relaxation of contracted rat aortic and human mesenteric arteries [28]. In order to investigate the specificity of the vasodilator effect of testosterone, we analysed the effect of cholesterol in rat aorta and in A7r5 cells. For the first time, we show that cholesterol, like testosterone, fully relaxed the arteries contracted by KCl, although the testosterone IC_50_ was bigger. Regarding the cholesterol effects on ionic channels, also for the first time, our results show that increasing concentrations of cholesterol inhibited basal and the BAY-stimulated I_Ca,L_, indicating a similareffect to that of testosteroneon these channels in rat aorta cells. Also, like testosterone, cholesterol failed to modify the potassium current measured by patch clamp. These results suggest that testosterone and cholesterol share the same mechanism in rat aorta, related with the inhibition of LTCC. 

 In summary, our results show that testosterone inhibits I_Ca,L_ in rat aorta vascular smooth muscle cells but not I_K_. Also, the effect of testosterone is not mediated by the classic hormone receptor or by potassium channel activation. In addition, our results show for the first time that cholesterol has similareffects to thattestosterone in rat aorta and in A7r5 cells, suggesting a common mechanism of action of both steroids that could be also shared by other steroids.

## Figures and Tables

**Table 1:** IC_50_ values (*μ*M) of testosterone relaxant effect on rat aortic rings contracted with BAY and with KCl (60 mM) alone or in the presence of the following drugs: the *K*_*V*_ channel blocker 4-aminopyridine (4-AP; 2 mM), the *B* *K*_*Ca*_ channel blocker tetraethylammonium (TEA; 1 mM), the *K*_*ATP*_ channel blocker glibenclamide (GLI; 10 *μ*M) and the testosterone receptor antagonists flutamide (10 *μ*M). Each value represents the mean ± SEM from the number of experiments shown in brackets. *P < .05 versus testosterone IC_50_ obtained in KCl-contracted arteries, Student's *t*-test.

Agents	Testosterone IC_50 _ (*μ*M)
BAY	12.94 ± 1.92 (*n* = 6)*
KCl	29.88 ± 1.12 (*n* = 5)
KCl + TEA	23.60 ± 1.06 (*n* = 4)
KCl + 4-AP	31.24 ± 1.15 (*n* = 5)
KCl + GLI	27.87 ± 1.21 (*n* = 3)
KCl + Flutamide	20.67 ± 1.26 (*n* = 4)

**Table 2:** Inhibitory effect of testosterone and cholesterol (1–100 *μ*M) on A7r5 cells basal potassium current (I_K_). Each value represents the mean of the % of variation of basal I_K_ ± SEM from the number of experiments shown in the brackets.

Concentration	Testosterone	Cholesterol
1 *μ*M	1.5 ± 1.9% (*n* = 10)	−1.8 ± 1.9% (*n* = 10)
10 *μ*M	−0.8 ± 2.3% (*n* = 7)	−1.3 ± 1.6% (*n* = 10)
30 *μ*M	−1.0 ± 1.7% (*n* = 9)	−0.0 ± 0.9% (*n* = 10)
100 *μ*M	−6.8 ± 3.3% (*n* = 9)	1.4 ± 0.8% (*n* = 8)
